# Esophageal Dieulafoy’s Lesion: A Rare and Life-Threatening Cause of Massive Upper Gastrointestinal Bleeding Successfully Treated With Argon Plasma Coagulation

**DOI:** 10.7759/cureus.82522

**Published:** 2025-04-18

**Authors:** Rim A Boutari, Zaher S Houmani, Yara S Tarhini, Ahmad G Mroue

**Affiliations:** 1 Gastroenterology and Hepatology, Al Zahraa Hospital University Medical Center, Beirut, LBN; 2 Internal Medicine, Al Zahraa Hospital University Medical Center, Beirut, LBN

**Keywords:** argon plasma coagulation, dieulafoy’s lesion, esophageal dieulafoy, hematemesis, hemostasis, upper gastrointestinal bleeding

## Abstract

Dieulafoy’s lesion is a rare but potentially life-threatening cause of upper gastrointestinal (UGI) bleeding. It is characterized by an abnormally large, tortuous artery in the submucosa that can erode the overlying mucosa and cause sudden, severe hemorrhage. While commonly found in the stomach, Dieulafoy's lesions in the esophagus are extremely rare and pose a diagnostic and therapeutic challenge. We report the case of a 23-year-old female with cerebral palsy, maintained on valproate and baclofen, who presented with multiple episodes of hematemesis. Laboratory findings revealed a hemoglobin level of 4 g/dL, significantly reduced from her baseline of 12 g/dL. After resuscitation with blood transfusions, the patient underwent urgent esophagogastroduodenoscopy, which identified an actively bleeding Dieulafoy’s lesion in the distal esophagus. Initial hemostatic clip placement failed, and hemostasis was successfully achieved using argon plasma coagulation (APC). The patient remained stable after the procedure and was discharged upon clinical improvement. Esophageal Dieulafoy’s lesion, though rare, should be considered in severe UGI bleeding. Endoscopic hemostasis is the mainstay of treatment, and APC can be effective when other methods fail.

## Introduction

Upper gastrointestinal (UGI) bleeding is a common medical emergency with various etiologies, ranging from peptic ulcer disease and variceal hemorrhage to less frequent vascular anomalies such as Dieulafoy’s lesion [1]. Dieulafoy’s lesion is a rare yet potentially fatal vascular anomaly characterized by a persistently dilated submucosal artery that erodes the overlying mucosa, causing sudden and severe hemorrhage. These lesions account for less than 5% of all gastrointestinal bleeding cases, highlighting their rarity but notable clinical significance [2]. Recent analyses, including a comprehensive review of 12,568 studies on gastrointestinal bleeding, have further emphasized the critical need to investigate and understand such rare causes [3]. While the stomach is the most common site, esophageal involvement is exceedingly rare, representing only a small proportion of non-variceal UGI bleeding cases [4].

Diagnosing esophageal Dieulafoy’s lesion is particularly challenging due to its episodic bleeding pattern and the absence of a typical ulcer or mass on endoscopy. Undiagnosed esophageal Dieulafoy's lesions carry a mortality risk of up to 6%, emphasizing the need for rapid recognition and timely intervention to prevent fatal outcomes [5]. Patients often present with massive hematemesis and hemodynamic instability, necessitating urgent resuscitation and endoscopic intervention [6]. Endoscopic therapy remains the cornerstone of treatment, with mechanical clipping, thermal coagulation, and injection therapy being first-line options. However, achieving hemostasis can be challenging, and in cases of treatment failure, alternative modalities such as argon plasma coagulation (APC) may be necessary [7].

Given the rarity of esophageal Dieulafoy’s lesions, reporting such cases is essential to enhance clinical awareness, refine diagnostic approaches, and optimize therapeutic strategies.

## Case presentation

A 23-year-old female with a history of cerebral palsy, maintained on valproate and baclofen, presented to the emergency department with multiple episodes of hematemesis and melena over the past 24 hours. Her family denied recent non-steroidal anti-inflammatory drug or anticoagulant use or a history of gastrointestinal bleeding. There was no prior diagnosis of liver disease, coagulopathy, or peptic ulcer disease.

On arrival, the patient appeared pale and lethargic, with tachycardia (HR: 120 bpm) and hypotension (BP: 85/50 mmHg), suggesting significant hemorrhagic shock. She was immediately resuscitated with intravenous fluids and received two units of uncrossmatched type O-negative packed red blood cells, given the urgency of the situation and the need for rapid blood replacement. This intervention resulted in transient hemodynamic improvement.

In addition, intravenous proton pump inhibitor (PPI) therapy was initiated with an 80 mg bolus of omeprazole, followed by a continuous infusion of 8 mg/hour to achieve rapid and sustained suppression of gastric acid secretion. This aimed to protect the mucosal lining and minimize the likelihood of further bleeding episodes.

Initial laboratory investigations revealed a hemoglobin level of 4.2 g/dL (baseline: 12 g/dL), hematocrit of 13%, normal platelet count (230,000/μL), and an international normalized ratio (INR) of 1.1, indicating no underlying coagulopathy. Liver function tests were within normal limits (Table 1).

**Table 1:** Laboratory findings on patient arrival INR: international normalized ratio, SGPT: serum glutamic pyruvic transaminase

Parameter	Value	Reference range
Hemoglobin	4.2 g/dL	12-16 g/dL
Hematocrit	13%	36-47%
Platelet count	230,000/μL	150,000-450,000/μL
INR	1.1	0.8-1.2
Creatinine	0.25 mg/dL	0.7-1.3 mg/dL
SGPT	35 U/L	7-56 U/L
Albumin	3.1 g/dL	3.5-5.0 g/dL

**Figure 1:**
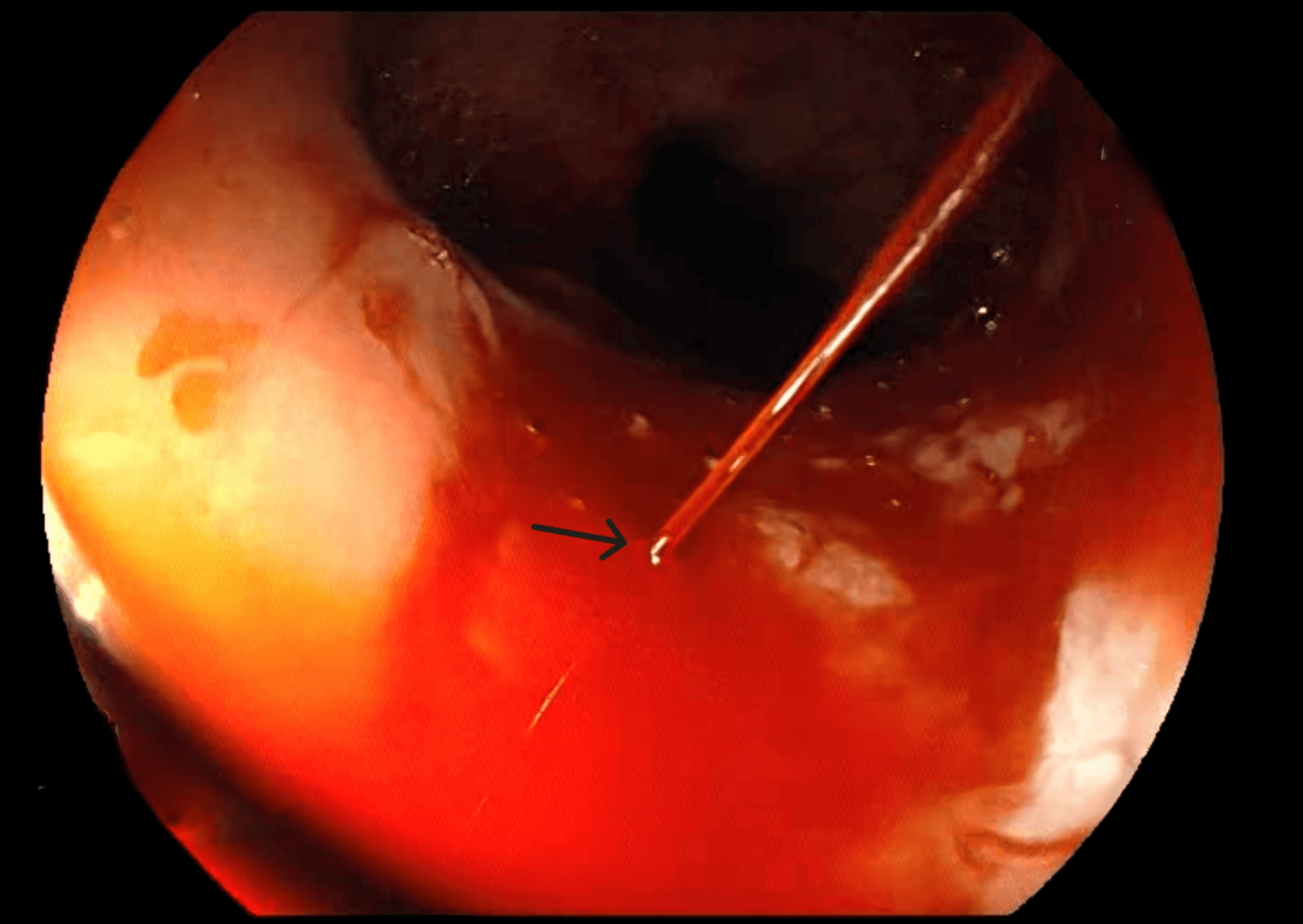
Actively bleeding Dieulafoy’s lesion in the distal esophagus Endoscopic view of an actively bleeding Dieulafoy's lesion in the distal esophagus. A prominent, aberrant submucosal artery is exposed with arterial spurting (indicated by the arrow), confirming the diagnosis.

Given the severity of bleeding, an urgent esophagogastroduodenoscopy was performed six hours after presentation. Endoscopic examination identified a 3 cm sliding hiatal hernia and an actively bleeding Dieulafoy’s lesion in the distal esophagus, characterized by a pinpoint mucosal defect with arterial spurting hemorrhage (Figure 1).

**Figure 2:**
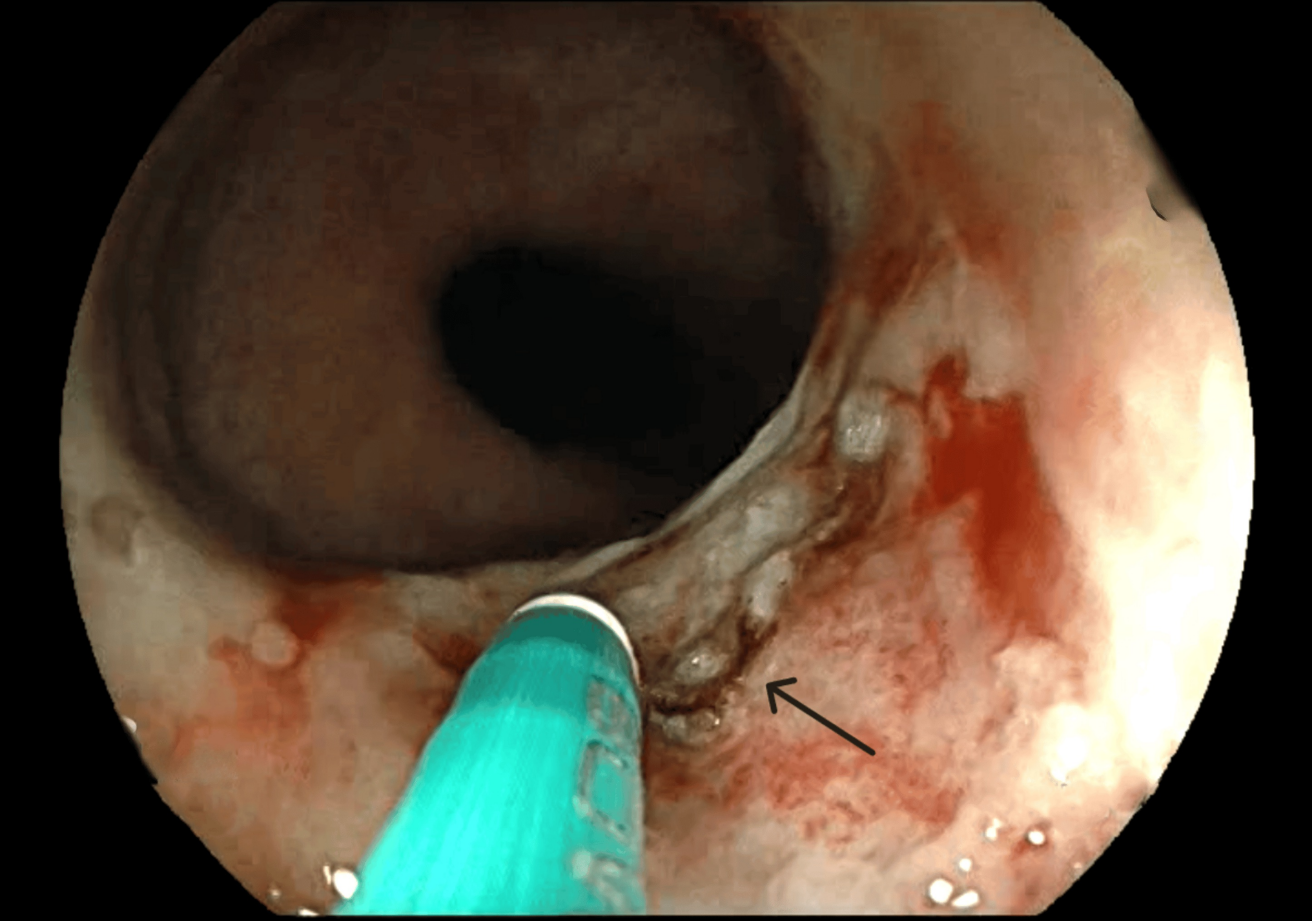
Hemostasis of esophageal Dieulafoy’s lesion using APC Endoscopic image demonstrating successful hemostasis of an esophageal Dieulafoy's lesion following APC. The previously bleeding aberrant submucosal artery (arrow) is now coagulated, with no active hemorrhage observed. Mucosal discoloration and coagulation marks indicate the site of thermal therapy. APC: argon plasma coagulation

No esophageal varices, ulcers, or additional sources of bleeding were detected. Hemostatic clip placement was attempted but failed due to the lesion’s difficult position, making adequate clip deployment challenging. Given ongoing bleeding, APC was applied, successfully achieving hemostasis with no immediate rebleeding (Figure 2).

Following the procedure, the patient was closely monitored in the hospital for 72 hours, with serial hemoglobin checks and hemodynamic assessment. She remained stable, with no further episodes of hematemesis. She was initiated on oral PPI therapy and was gradually transitioned to oral intake. After achieving clinical stabilization, the patient was discharged in stable condition. An outpatient follow-up was scheduled to include surveillance endoscopy and further evaluation of potential risk factors for recurrent bleeding.

**Figure 3:**
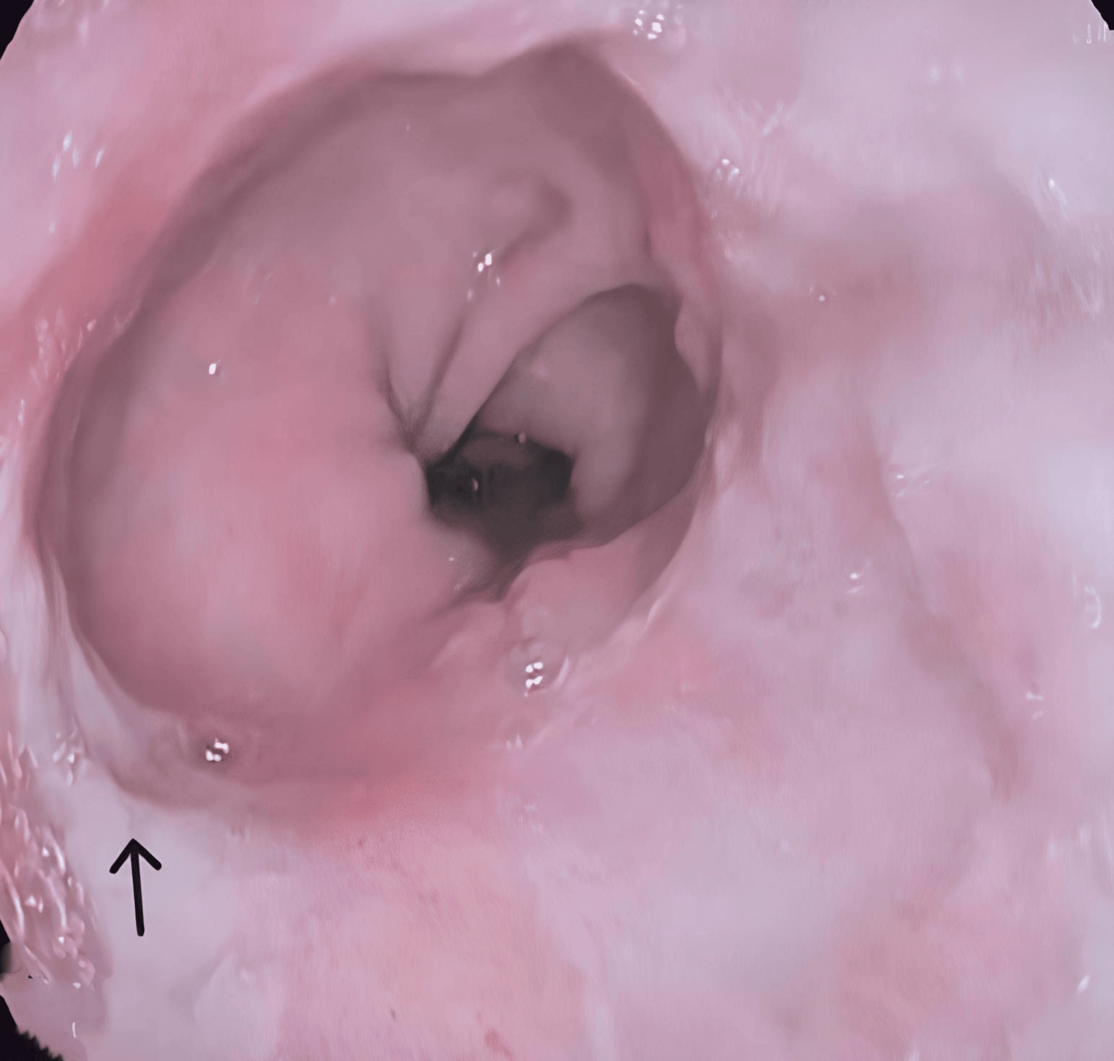
Endoscopic appearance of esophageal mucosa three weeks post APC for Dieulafoy’s lesion Follow-up endoscopic image showing the esophageal mucosa three weeks after APC treatment for a Dieulafoy's lesion. The previously treated site (arrow) appears well-healed, with no evidence of recurrent bleeding or mucosal ulceration, indicating successful hemostasis and tissue recovery. APC: argon plasma coagulation

Three weeks following APC treatment, a follow-up gastroscopy was performed. The examination revealed normal esophageal mucosa with no signs of bleeding, indicating that APC is an effective treatment for Dieulafoy’s lesions, achieving successful hemostasis (Figure 3).

## Discussion

Esophageal Dieulafoy’s lesion is an extremely rare but potentially fatal cause of massive UGI bleeding, often presenting with sudden and severe hematemesis [2]. Unlike peptic ulcers or varices, Dieulafoy’s lesions arise from an abnormally large submucosal artery that erodes through the mucosa, leading to arterial hemorrhage. These lesions are classically found in the stomach, predominantly on the lesser curvature, but up to one-third may occur outside the stomach, most commonly in the duodenum and colon [8]. Although typically presenting with massive hematemesis, some cases exhibit intermittent, self-limiting bleeding, which can delay diagnosis. Given its unpredictable bleeding pattern, prompt resuscitation and early endoscopy are crucial.

The exact etiology of Dieulafoy’s lesions remains uncertain, with both congenital and acquired mechanisms proposed. Some studies suggest a congenital origin due to an abnormally dilated submucosal artery. In contrast, others point to acquired factors such as chronic mucosal injury from gastroesophageal reflux disease, peptic ulcer disease, or the use of nonsteroidal anti-inflammatory drugs [8]. In our patient, the presence of a hiatal hernia may have predisposed them to chronic mucosal irritation, which was further exacerbated by baclofen use. Baclofen is known to relax the lower esophageal sphincter and worsen gastroesophageal reflux.

Diagnosing esophageal Dieulafoy’s lesions is challenging due to their intermittent bleeding pattern and the absence of a visible ulcer or mass. While endoscopy remains the gold standard, lesions may be missed if bleeding has ceased at the time of the procedure [9]. In such cases, repeat endoscopy or adjunctive imaging, such as CT angiography, may be necessary. Capsule endoscopy or deep enteroscopy can also be considered if there is suspicion of more distal small bowel involvement [9].

Endoscopic therapy is the mainstay of treatment, with mechanical clips, thermal coagulation, and injection therapy being commonly used [10]. In our case, initial hemostatic clipping failed due to the lesion’s location, but APC successfully controlled the bleeding. While APC and clipping are widely used, alternative endoscopic techniques have been explored. A study by Barakat et al. (2018) compared the effectiveness of endoscopic hemoclip placement versus band ligation, suggesting that band ligation may be a viable option in selected cases, particularly for lesions with a well-defined feeding artery [11]. In refractory cases, newer modalities such as over-the-scope clips or hemostatic powders have shown promise. At the same time, angiographic embolization or surgical resection may be required for patients with persistent or recurrent bleeding [7].

Despite successful hemostasis, Dieulafoy’s lesions carry a risk of rebleeding, which is reported in up to 9% to 40% of cases. Close post-procedural monitoring is essential, and patients should be evaluated for underlying risk factors that may contribute to recurrence [10]. Long-term management strategies include PPI therapy, particularly in cases associated with gastroesophageal reflux, as well as lifestyle modifications to reduce mucosal injury. Although rare, esophageal Dieulafoy’s lesions should be considered in patients with unexplained severe UGI hemorrhage, as timely recognition and appropriate management are crucial for improving outcomes [12].

## Conclusions

Esophageal Dieulafoy’s lesion is a rare yet serious cause of UGI bleeding that requires rapid recognition and intervention to prevent life-threatening complications. Its intermittent bleeding pattern and the absence of distinct endoscopic findings make the diagnosis challenging, underscoring the need for a high degree of clinical suspicion and early endoscopic evaluation. Endoscopic therapy is the mainstay of treatment, but no single modality is universally effective, necessitating a tailored approach based on lesion characteristics and bleeding severity. In our case, APC successfully achieved hemostasis after initial clip failure, demonstrating its efficacy as a salvage option. Emerging techniques, such as over-the-scope clips and band ligation, offer promising alternatives for refractory cases. Close post-procedural monitoring and long-term strategies, including acid suppression and modifying risk factors, remain vital to improving patient outcomes and reducing the risk of rebleeding. Enhanced awareness of this rare entity will contribute to advancements in diagnostic and therapeutic approaches, ensuring better management of future cases.
